# Effects of Rumen-Protected 5-Hydroxytryptophan and Melatonin Supplementation on Antioxidant Capacity, Meat Quality, and Shelf Life of Hu Sheep

**DOI:** 10.3390/ani15111582

**Published:** 2025-05-29

**Authors:** Shiheng Xu, Honghai Yang, Zulibina Ainiwaer, Wenpeng Fan, Tongxiang Xu, Kailun Yang, Caidie Wang

**Affiliations:** Xinjiang Key Laboratory of Herbivore Nutrition for Meat & Milk, Research Center for Biofeed and Animal Gut Healthz, College of Animal Sciences, Xinjiang Agricultural University, Urumqi 830052, China; 18160220952@163.com (S.X.);

**Keywords:** antioxidation, Hu sheep, meat quality, melatonin, shelf life, 5-hydroxytryptophan

## Abstract

This study aimed to investigate the impact of dietary supplementation with RPT 5-HTP and MT on the meat quality and shelf life of Hu sheep. Thirty 4-month-old male Hu sheep with similar body weights (30.84 ± 1.16 kg) were randomly allocated into three groups (one sheep per replicate): a control group (fed a basal diet), a RPT 5-HTP group (basal diet + 222 mg/kg BW RPT 5-HTP), and an MT group (basal diet + 4 mg/d MT). The experimental period lasted 37 days, including a 7-day adaptation phase and a 30-day formal trial. The results show that supplementation with RPT 5-HTP and MT significantly increased MT levels in the *longissimus dorsi* muscle of Hu sheep (*p* < 0.05). Compared to the control group, the malondialdehyde (MDA) content in the *longissimus dorsi* muscle was significantly reduced in the MT and RPT 5-HTP groups (*p* < 0.05), while catalase (CAT) activity was significantly increased in the MT group (*p* < 0.05). The drip loss of the *longissimus dorsi* muscle in Hu sheep was significantly reduced after supplementation with RPT 5-HTP and MT (*p* < 0.05). Essential amino acids, non-essential amino acids, sweet-tasting amino acids, and umami amino acids in the *longissimus dorsi* muscle of supplemented groups were significantly elevated (*p* < 0.05). Both 5-HTP and MT extended the shelf life of mutton; compared to the control, the shelf life was prolonged by 22.08 h in the RPT 5-HTP group and by 45.36 h in the MT group. In conclusion, it was found that 5-HTP and MT had a certain effect on improving the antioxidant capacity of Hu sheep, thereby enhancing the meat quality of mutton and extending its shelf life, with the amino acid content in mutton being increased to improve its umami and flavor.

## Simple Summary

The antioxidant capacity of meat products is inherently linked to their shelf life and quality attributes. Melatonin (MT) and 5-hydroxytryptophan (5-HTP) are well known for their strong antioxidant properties. Direct MT supplementation may potentially inhibit endogenous MT synthesis, thereby reducing systemic MT levels in animals. Consequently, this study investigates the effects of rumen-protected 5-hydroxytryptophan (RPT 5-HTP) and melatonin (MT) supplementation on mutton quality and shelf life. The results show that all experimental groups reduced the drip loss of mutton. The RTP 5-HTP group reduced it by 18.58%, and the MT group reduced it by 33.87%. Additionally, both groups prolonged the shelf life of mutton, with the RTP 5-HTP group extending it by 22.08 h and the MT group by 45.36 h.

## 1. Introduction

Sheep meat is regarded as a functional food due to its high nutritional properties, including a high content of essential amino acids and its sanogenic lipid profile [1]. Hu sheep possess excellent traits, such as producing two litters per year with multiple lambs per litter, rapid growth and development, high tolerance to heat and humidity, and ideal meat production performance after improvement. However, the consumption of sheep meat is often constrained by its off-flavor and limited shelf life, resulting in reduced market value [2]. Therefore, the extension of shelf life and assurance of meat quality are considered particularly important. Meat quality is significantly influenced by the oxidative status of muscle tissue, which is correlated with the content or activity of endogenous antioxidants in the body. The improvement in meat quality and extension in shelf life can be achieved by enhancing muscular antioxidant capacity [3]. Melatonin (MT) is recognized as possessing strong antioxidant properties and is widely present in mammals as an endogenous hormone.

MT, scientifically termed N-acetyl-5-methoxytryptamine, was first discovered in the late 1950s [4]. MT is classified as an indoleamine compound that is predominantly synthesized and secreted by the pineal gland, while additional secretion is observed in the retina, accessory lacrimal glands, and gastrointestinal tract [5]. Significant regulatory functions are exerted by MT in various physiological processes, including circadian rhythm modulation [6], immune competence maintenance [7], antioxidant defense [8], and lipid metabolism regulation [9]. In plants, MT is utilized as an antioxidant through which the metabolic balance of reactive oxygen species (ROS) is maintained [10], while fruit quality is enhanced and decay is delayed [11]. In animals, the primary physiological function of MT is considered to be the transmission of circadian cycle information to signal receptors in the brain [12]. In livestock production, MT has been demonstrated to regulate reproductive functions [13]; improve cashmere fiber length, fineness, and density in goats [14]; and promote growth and development in lambs [15]. MT and 5-hydroxytryptophan (5-HTP) are recognized for their potent antioxidant properties through which hydroxyl radicals and ROS are scavenged, while the activity and expression of endogenous antioxidant enzymes are enhanced, thereby improving overall antioxidant capacity [16]. MT has also been shown to activate mitochondrial nitric oxide synthase while inhibiting cerebellar nitric oxide synthase activity, leading to reduced nitric oxide production [17]. In the study conducted by Gonzalez-Candia et al., it was demonstrated that prenatal supplementation with MT (10 mg/d) in pregnant ewes significantly enhanced the antioxidant capacity of newborn lambs, while the production of pro-oxidant ROS was reduced [18]. Choudhary et al. [19] demonstrated that supplementation with MT (3 mg/d) in ewes significantly decreased the blood malondialdehyde (MDA) content and increased superoxide dismutase (SOD) and catalase (CAT) activities during the first month of the experiment. The aforementioned studies demonstrated that the antioxidant capacity of animal organisms can be enhanced by MT. It has been established in existing research that the oxidative deterioration of meat can be significantly reduced and the shelf life can be extended through the addition of natural antioxidants to meat products and through dietary supplementation with substances possessing antioxidant properties [20]. Therefore, a hypothesis was proposed that meat quality and shelf life might be improved through the elevation of MT levels in sheep. The effects of supplementation with rumen-protected 5-hydroxytryptophan (RPT 5-HTP) and MT on the meat quality and shelf life of Hu sheep mutton were investigated in this experiment, and references were provided for the application of 5-HTP and MT in improving mutton quality and extending shelf life.

## 2. Materials and Methods

### 2.1. Ethical Considerations

This study was approved by the Animal Welfare and Ethics Committee of Xinjiang Agricultural University (Animal protocol number: 2023011).

### 2.2. Materials

MT purity ≥ 98% (Sigma-Aldrich Trading Co., Ltd., Shanghai, China); 5-HTP purity 98% (Wuhan Yuancheng Co-creation Technology Co., Ltd., Wuhan, China). RPT 5-HTP was manufactured by Beijing Yahe Nutrition High-Tech Co., Ltd. (Beijing, China), using the aforementioned 5-HTP as raw material, with an effective 5-HTP content of 45.00% and a rumen bypass rate of 88.60%; 5-HTP is rumen-protected, whereas MT is untreated. C16-C18 medium-chain fatty acids served as the rumen protection material.

### 2.3. Diets, Experimental Design, and Animal Management

Thirty healthy 4-month-old Hu sheep rams with similar body weights (30.84 ± 1.16 kg) were randomly assigned to three groups with ten replicates in each group (one sheep per replicate). All experimental sheep were fed the same basal diet with the same nutritional level (see Table 1). On this basis, the RPT 5-HTP group was supplemented with RPT 5-HTP at 222 mg/(kg·BW) per day [21], and the MT group was supplemented with MT at 4 mg/sheep [22]. The sheep in the RPT 5-HTP group were weighed every day and evenly divided into two portions. MT was dissolved in an appropriate amount of ethanol, and the volume was made up to 50 mL with distilled water. The solution was then transferred to a brown bottle and stored in a refrigerator at 4 °C. In the RPT 5-HTP group, one portion of RPT 5-HTP was supplemented in the morning and the other in the evening every day. In the MT group, 1 mL (containing 2 mg of MT) of MT solution was supplemented in the morning and evening every day. The experimental period was set at 37 days, consisting of a 7-day adaptation period followed by a 30-day formal trial period.

The feeding trial was carried out at the sheep farm of the Xinjiang Tianshan Huangniu Breeding Base. Feeding operations were conducted at 10:30 a.m. and 7:00 p.m. daily. Prior to feeding, 2 kg of the mixed diet was mixed with a small amount of water. In the control group, 50 g of feed was provided to each sheep. For the experimental groups, pre-weighed RPT 5-HTP and MT solutions were thoroughly mixed with 50 g of the mixed diet before being administered. Feeding was performed twice daily (morning and evening). After complete consumption was observed, the remaining basal diet was weighed and subsequently provided. All experimental sheep were housed in individual pens with free access to water and feed. Regular cleaning and disinfection of pens were performed according to the farm’s management protocols.

### 2.4. Growth Performance

During the experimental period, the weights of the daily feed ration and the residual feed from the previous day for each Hu sheep were recorded before morning feeding, and the body weight of each Hu sheep was measured before morning feeding on day 1 and day 28 of the experiment. Data were used for calculating the average daily gain (ADG), dry matter intake (DMI), and the ratio of feed intake to gain (F/G).

Dry matter intake (DMI, kg/d) was calculated as follows:

DMI = Total dry matter intake (kg)/Number of experimental days (d).

Average daily gain (ADG, g) was determined as follows:

ADG = [Final body weight (kg) − Initial body weight (kg)]/Number of experimental days (d) × 1000.

Feed intake to gain (F/G) was defined as follows:

F/G = Dry matter intake (g/d)/Average daily gain (g/d).

### 2.5. Slaughter Performance and Muscle Sampling

Upon the termination of the experimental period, the sheep were subjected to a 12 h pre-slaughter fasting period, with water access being restricted during the final 2 h preceding slaughter, and six sheep were selected from each group for slaughter. The slaughtering procedures were performed according to the regulation of the industrial and sanitary inspection of animal products of the Ministry of Agriculture, Livestock, and Supply. After slaughter, the carcass weight was measured, and the Greyhound Rib (GR) value and eye muscle area were determined. The muscle tissue was collected from the *longissimus dorsi* muscle at the 12th–13th lumbar vertebrae on the left side of the sheep to determine meat quality. Meanwhile, an appropriate amount of *longissimus dorsi* muscle at the 12th–13th lumbar vertebrae on the other side was taken, trimmed of fascia, divided into portions, and stored in 5 mL cryogenic tubes at −20 °C and then brought back to the laboratory for the analysis of the MT content, antioxidant capacity, amino acids, fatty acids, and shelf life.

Carcass weight: Carcass weight is defined as the weight (in kilograms, kg) recorded after slaughter with the wool, hide, feet, and viscera removed (except for the kidneys and perirenal fat).

GR value: The tissue thickness (mm) between the 12th and 13th ribs and at 11 cm from the dorsal midline was measured with Vernier calipers.

Eye muscle area: The cross-sectional area of the *longissimus dorsi* muscle over the lumbar vertebrae between the 12th and 13th ribs was measured, and the eye muscle area was calculated using the following formula:

Eye muscle area = Eye muscle height × Eye muscle width × 0.7 (cm).

### 2.6. Sample Preparation and Determination

#### 2.6.1. Determination of MT Content

The samples of *longissimus dorsi* muscle were sent to the Beijing SINO-UK Institute of Biological Technology, Research Institute to determine the content of MT using a Huawei Delang DR-200BS enzyme-labeled analyzer (Wuxi Huawei Delang Instrument Co., Ltd., Wuxi, Jiangsu, China).

#### 2.6.2. Determination of Antioxidant Activity

Sample Pretreatment: The *Longissimus dorsi* muscle samples stored at 4 °C were accurately weighed, and pre-cooled physiological saline was added at a ratio of 1:10 (*m*/*v*). The mixture was homogenized using a high-speed homogenizer and centrifuged at 2500 r/min for 10 min, and the supernatant was collected for subsequent analysis.

Determination of Antioxidant Parameters: Total antioxidant capacity (T-AOC), glutathione peroxidase (GSH-Px), total superoxide dismutase (T-SOD), malondialdehyde (MDA), and catalase (CAT) contents in the *Longissimus dorsi* muscle of experimental sheep were determined using commercial assay kits, with samples analyzed by the Nanjing Jiancheng Bioengineering Institute.

#### 2.6.3. Determination of Meat Quality

The pH values of the *Longissimus dorsi* muscle were measured at 45 min and 24 h postmortem using a portable pH meter (Shanghai Detu Instruments International Trade Co., Ltd., Shanghai, China). A cross-shaped incision was created on the muscle surface to insert the glass electrode, and triplicate measurements per sample were recorded after stabilization. The meat color was determined using a JZ-350 portable color difference meter (Shanghai Shouli Industrial Co., Ltd., Shanghai, China). The lightness (L*), redness (a*), and yellowness (b*) of the meat samples were measured. Three replicates were performed for each meat sample, and the average value was calculated. For drip loss measurement, the sample was cut into a muscle block with dimensions of 3 cm × 2 cm × 2 cm and weighed (W1). One end of the trimmed lamb sample was suspended in a measuring plastic bottle without touching the bottle wall. The sample was placed in a refrigerator at 4 °C for 24 h. After removal, the surface moisture of the meat sample was wiped off with filter paper, and the sample was weighed again (W2). The drip loss rate was calculated using the following formula: Drip loss rate (%) = [(W1 − W2)/W1] × 100%. For the determination of cooking loss, the peripheral muscle membrane of the meat sample was removed. A piece of meat sample weighing approximately 15 g was taken, numbered, and accurately weighed (m1). It was then placed in a self-sealing bag. The self-sealing bag was put into a constant-temperature water bath at 80 °C for 30 min. After removal, it was allowed to cool to room temperature, the surface moisture was drained, and the sample was weighed again (m2). The cooking loss was calculated using the following formula: Cooking loss (%) = [(m1 − m2)/m1] × 100%. For shear force measurement, for the meat samples used for cooking loss determination, a circular sampler was used to collect samples along the direction of muscle fibers. The shear force was measured using a C-LM3B digital muscle tenderness tester (Tenovo International Co., Ltd., Beijing, China). Three replicates were conducted for each meat sample, and the average value was calculated.

#### 2.6.4. Determination of Amino Acids and Fatty Acids

The content of amino acids and fatty acids in the *longissimus dorsi* muscle was analyzed and detected by high-performance liquid chromatography–tandem mass spectrometry (HPLC-MS/MS) by Puri Huasheng Medical Inspection Co., Ltd. (Tianjin, China). The instrument model used was Shimadzu LC20AD-API 3200MD TRAP (Shimadzu Enterprise Management Co., Ltd., Shanghai, China).

Liquid chromatographic conditions for amino acids are as follows: MSLAB − 45 + AA (Tianjin Purui Huasheng Medical Laboratory Co., Ltd., Tianjin, China). Methanol, acetonitrile, etc., were all purchased from Sigma-Aldrich Corporation (St. Louis, MO, USA) in the United States. Chromatographic column: Venusil MP C18 (150 × 4.6 mm, 5 μm). Column temperature: 50 °C. Flow rate: 1 mL/min. Mobile phase A (organic phase): Acetonitrile (containing 0.1% formic acid and 0.01% heptafluorobutyric acid); mobile phase B (aqueous phase): water (containing 0.1% formic acid and 0.01% heptafluorobutyric acid). Injection volume: 3 μL. Mass spectrometric conditions are as follows: The ion source is an ESI positive electrospray ionization source. Multiple reaction monitoring (MRM) was adopted. Curtain gas (CUR): 20 psi. Spray voltage (IS): +5500 V. Collision gas (CAD): medium. Collision cell exit potential (CXP): 2.0. Nebulizer gas (GS1): 55 psi. Auxiliary gas (GS2): 60 psi. Nebulizer temperature (TEM): 500 °C. Ion spray voltage (EP): 10 V.

Fatty acid GC conditions are as follows: column: Agilent G3903-63011, DB-FastFAME; injection port temperature: 250 °C; split ratio: 10:1; injection volume: 1 μL; carrier gas: helium; temperature program: initial column temperature of 50 °C held for 0.5 min, increased at a rate of 35 °C/min to 194 °C and held for 3.5 min, and then ramped at a rate of 9 °C/min to 240 °C and maintained for 1 min.

#### 2.6.5. Determination of Shelf Life

We accurately weighed 5 g of lamb sample rounded to the nearest 0.001 g and placed it in a conical flask. We added 37.5 mL of distilled water to the conical flask. We homogenized the sample using a high-speed homogenizer to ensure uniform dispersion of the sample in the solution. After standing for 30 min, we filtered the sample and stored it in a volumetric flask. We determined the total volatile basic nitrogen (TVB-N) content in the lamb according to the semi-micro Kjeldahl method specified in the national standard GB 5009.228-2016 [23] for the determination of volatile basic nitrogen in food.

By determining the TVB-N content in meat samples stored at 4 °C at different time intervals (1, 2, 4, 6, 8, 10, and 12 days), and taking the time when TVB-N reaches 15 mg/100 g as specified in the national food safety standard GB 2707-2016 [24] as the standard, the linear equations of TVB-N content for the control group, RPT 5-HTP group, and MT group were obtained through the standard curve. Subsequently, the specific time for the TVB-N value of each group to reach 15 mg/100 g was further calculated.

### 2.7. Statistical Analysis

The experimental data were initially organized using Microsoft Excel 2016. Statistical analysis was performed with IBM SPSS Statistics 26 (SPSS, Inc., Chicago, IL, USA), employing a one-way analysis of variance (ANOVA) followed by Duncan’s multiple range test for post hoc comparisons. Results are expressed as mean and standard error of the mean (SEM), with differences considered statistically significant at *p* < 0.05.

## 3. Results

### 3.1. Effects of RPT 5-HTP and MT Supplementation on Growth Performance of Hu Sheep

As shown in Table 2, supplementation with 5-HTP and MT in the diet had no significant effects on the final body weight, ADG, DMI, and F/G in Hu sheep (*p* > 0.05).

### 3.2. Effects of RPT 5-HTP and MT Supplementation on Slaughter Performance of Hu Sheep

As shown in Table 3, no significant differences in the GR and eye muscle area were detected between the 5-HTP and MT groups of Hu sheep compared to the control group (*p* > 0.05). While the MT group displayed numerically higher GR and eye muscle area values, these differences did not attain statistical significance. No significant differences were observed in live weight before slaughter, carcass weight, or dressing percentage among the groups of Hu sheep (*p* > 0.05).

### 3.3. MT Content

**Figure 1:**
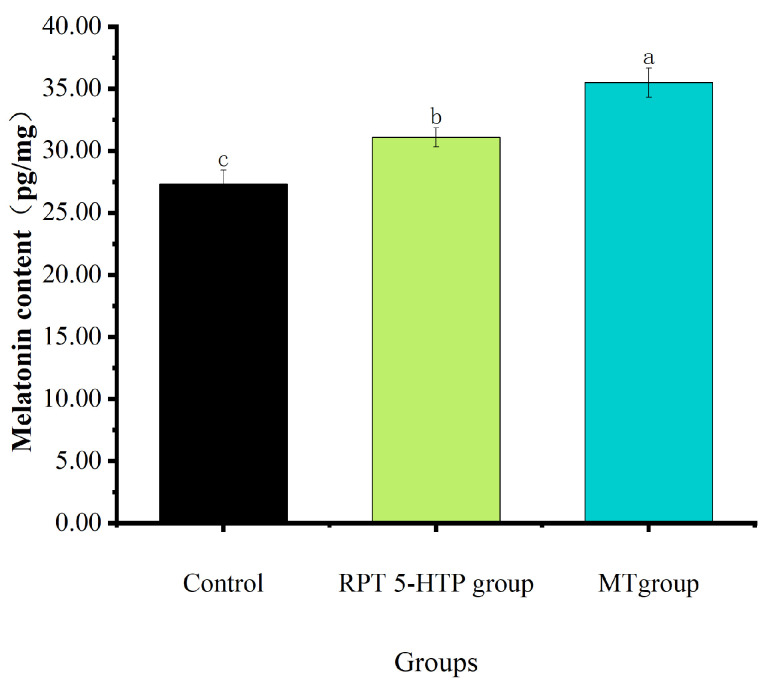
MT content in *longissimus dorsi* muscle of Hu sheep. Different letters indicate significant difference (*p* < 0.05).

As shown in Figure 1, compared with the control group, supplementation with RPT 5-HTP and MT significantly increased the MT content in the *longissimus dorsi* muscle of Hu sheep (*p* < 0.05).

### 3.4. Antioxidation

As shown in Table 4, the MDA content in the *longissimus dorsi* muscle was significantly lower in the 5-HTP and MT groups compared to the control group (*p* < 0.05), and it was reduced by 22.25% and 22.74%, respectively. The MT group showed significantly higher CAT activity in the *longissimus dorsi* muscle than the control group (*p* < 0.05), as it increased by 28.21%, whereas no significant difference in CAT activity was observed between the RPT 5-HTP group and the control group (*p* > 0.05). No statistically significant differences in SOD activity, GSH-Px activity, or T-AOC were detected between the treatment groups and the control group (*p* > 0.05).

### 3.5. Meat Quality

As shown in Table 5, the drip loss in the *longissimus dorsi* muscle of Hu sheep was significantly lower in the MT and RPT 5-HTP groups compared to the control group (*p* < 0.05), and the values were reduced by 18.58% and 33.87%, respectively. No statistically significant differences (*p* > 0.05) were observed among the groups for pH_45min_, pH_24h_, L*, a*, b*, shear force, and cooking loss.

### 3.6. Amino Acid

As shown in Table 6, compared with the control group, both 5-HTP and MT supplementation significantly increased serine (118.67% and 194.30%), valine (18.00% and 30.00%), threonine (30.64% and 59.15%), isoleucine (19.81% and 31.76%), phenylalanine (18.63 and 28.80%), alanine (28.84% and 48.59%), tyrosine (22.26% and 32.94%), proline (22.24% and 35.43%), glutamic acid (25.20% and 51.58%), and methionine (22.85% and 39.31%) contents in the *longissimus dorsi* muscle of Hu sheep (*p* < 0.05). Among these, the MT group exhibited significantly higher threonine and glutamic acid levels than the RPT 5-HTP group (*p* < 0.05), and the values were increased by 21.82% and 21.06%, respectively. Additionally, the MT group showed significantly higher serine, valine, isoleucine, alanine, proline, and methionine contents compared to the RPT 5-HTP group (*p* < 0.05), which were higher by 34.59%, 10.17%, 9.10%, 15.33%, 1.79%, and 13.39%, respectively. The leucine, histidine, and arginine contents in the *longissimus dorsi* muscle were significantly higher in the MT group than in the control group (*p* < 0.05) by 30.22%, 17.88%, and 20.76%, respectively, while the RPT 5-HTP group displayed a significantly higher leucine content than the control group (*p* < 0.05), showing an increase of 17.93%. The histidine content in the MT group was also significantly elevated compared to the control (*p* < 0.05). No significant differences in the tryptophan, lysine, glycine, or aspartic acid contents were observed among the treatment groups (*p* > 0.05). Furthermore, the RPT 5-HTP group and MT group demonstrated significantly higher contents of essential amino acids (15.22% and 28.29%), non-essential amino acids (22.85% and 43.39%), sweet-tasting amino acids (18.68% and 35.81%), and umami-tasting amino acids (27.88% and 48.45%) in the *longissimus dorsi* muscle compared to the control (*p* < 0.05). The contents of essential amino acids, non-essential amino acids, sweet-tasting amino acids, and umami-tasting amino acids in the *longissimus dorsi* muscle of Hu sheep in the MT group were significantly higher than those in the RPT 5-HTP group (*p* < 0.05) by 11.35%, 16.72%, 14.43%, and 16.08%, respectively.

### 3.7. Fatty Acid

As shown in Table 7, the stearic acid (C18:0) content in the *longissimus dorsi* muscle of the RPT 5-HTP group of Hu sheep was significantly lower than that in the control group (*p* < 0.05), showing a reduction of 52.35%. The oleic acid (C18:1n9c) and eicosadienoic acid (C20:2) contents in the *longissimus dorsi* muscle of the MT group of Hu sheep were also significantly lower than those in the control group (*p* < 0.05) by 47.94%. No significant differences in other fatty acid contents were observed among the groups (*p* > 0.05). No significant differences in saturated fatty acid (SFA) and polyunsaturated fatty acid (PUFA) contents were detected between the treatment groups and the control group (*p* > 0.05). However, the monounsaturated fatty acid (MUFA) content in the *longissimus dorsi* muscle of the 5-HTP and MT groups of Hu sheep was significantly lower than that in the control group (*p* < 0.05), which decreased by 39.11% and 46.60%, respectively.

### 3.8. Shelf Life

**Figure 2:**
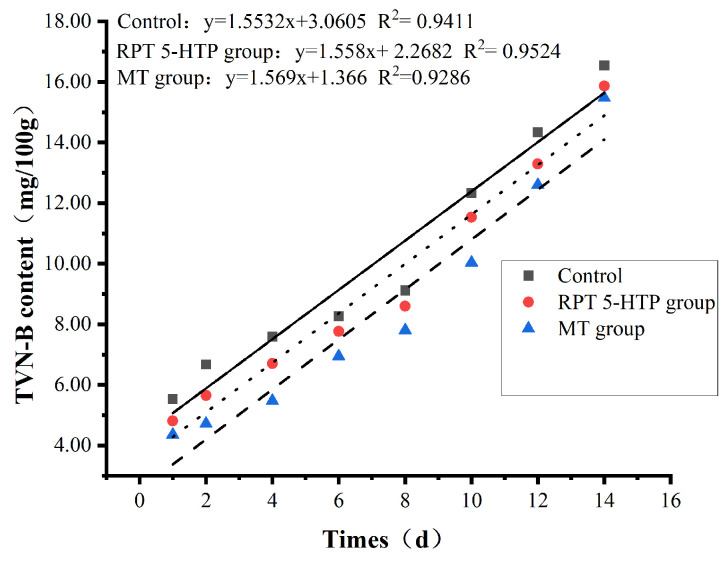
Effects of 5-HTP and MT supplementation on shelf life of *longissimus dorsi* muscle of Hu sheep.

Figure 2 illustrates the changes in the TVB-N content in the *longissimus dorsi* muscle among groups. The results show that both the 5-HTP and MT groups reduced the TVB-N content in the longest dorsal muscle of lake sheep compared with the control group. The calculated values show that the time required for the TVB-N content to reach 15 mg/100 g was 13.21 days (control group), 14.13 days (RPT 5-HTP group), and 15.10 days (MT group). Compared to the control group, the 5-HTP and MT groups prolonged the shelf life by 22.08 h and 45.36 h, respectively.

## 4. Discussion

The eye muscle area serves as a critical indicator in animal production, demonstrating a strong correlation with meat production performance and carcass quality; it can be utilized to distinguish superior breeds within the same species through comparative analysis [25]. In this experiment, no significant differences were observed in the eye muscle area among all groups. And there were no significant differences in carcass weight, slaughter rate, and GR among different groups of Hu sheep. Sahin et al. [26] reported that MT supplementation in quail diets did not significantly influence the final weight and carcass weight. Similarly, MT implants (2 mg/kg BW) showed no significant impact on the ADG, hot carcass weight, and eye muscle area in cashmere goats [27]. Elevated MT levels in broilers under varying light conditions also failed to influence body weight [28]. These findings align with the results of the present study, confirming that MT has no significant effect on slaughter performance in animals. In contrast, other studies have shown that pinealectomy significantly reduces the MT concentration in broilers, while exogenous MT supplementation increases body weight [29]. Sun et al. [30] found that when 5-HTP was added to the diet of sheep, the body weight of the 5-HTP-supplemented group significantly increased on day 30 of the experiment, and the average daily gain at each growth stage significantly increased. This result is different from the findings of the present study, likely due to variations in experimental methods and feeding supplementation levels. The above research indicates that the effects of MT or 5-HTP treatment on body weight and slaughter performance vary among different animals.

In mammals, MT is primarily synthesized in the pineal gland and intestines, with minor contributions from other tissues and organs [31]. The secretion of MT from the pineal gland and retina exhibits circadian rhythmicity, characterized by lower daytime levels and higher nocturnal concentrations [32]. Early studies proposed two methods to regulate the MT content in animals: direct intravenous injection or dietary supplementation [33] and oral or injectable administration of MT precursors (e.g., tryptophan and 5-HTP) [34]. Research indicates that under a 12 h light/dark (12 L:12 D) cycle, the MT concentrations in sheep have a range of 10–30 pg/mL during the day and 100–300 pg/mL at night [35]. Namboodiri et al. [36] demonstrated that exogenous MT or its precursors elevated plasma MT levels in sheep. For instance, the intraperitoneal injection of 20 or 200 mg/kg BW 5-HTP at 07:00 resulted in plasma MT concentrations of 64.32 and 349.89 pg/mL at 12:00, respectively, significantly higher than the control group (23 pg/mL). Supplementation with 111 or 222 mg/kg BW RPT 5-HTP increased daytime and nocturnal plasma MT concentrations in sheep [21]. A single intraperitoneal injection of 20 mg/kg BW 5-HTP caused a rapid MT surge, peaking at 159 pg/mL 2 h post-injection [37]. Salamanders were administered 5-HTP via intraperitoneal injection at doses of 10, 20, and 40 mg/kg. In the 40 mg/kg group, the plasma MT concentrations were significantly elevated and peaked 4 h post-injection. For the 10 and 20 mg/kg groups, plasma MT concentrations significantly increased and reached peak levels 8 h post-injection [38]. The plasma MT concentration significantly increased after sheep were orally administered 1 mg/kg MT for 8 consecutive days [39]. Pan et al. [40] found in their study on duodenal 5-HTP infusion in sheep that the MT content in the entire intestinal mucosa of sheep significantly increased in both the 10 mg and 50 mg 5-HTP infusion groups, and the MT content in plasma significantly increased. Building on prior research, this study employed oral supplementation and achieved results consistent with existing evidence. These studies show that injecting MT, supplementation with RPT 5-HTP, and the oral perfusion of 5-HTP in sheep significantly increased the MT levels in animal bodies. This study refers to Zhao et al., who significantly increased the MT content in the *longissimus dorsi* muscle of Hu sheep by providing supplementary RPT 5-HTP through feeding.

Animals are exposed to oxidative stressors (endogenous, exogenous, and damaging factors) during metabolic processes. MT, as an endogenous antioxidant, exhibits unique antioxidative effects distinct from classical antioxidants [41]. On the one hand, MT acts as an effective antioxidant that delays lipid and protein oxidation in meat, potentially extending the shelf life while preserving flavor and texture [42]. On the other hand, the antioxidant enzyme system comprising SOD, GSH-Px, and CAT is enhanced by exogenous MT, which elevates enzyme activities to scavenge free radicals and reduce ROS and MDA levels. In our study, the content of MDA in the *longissimus dorsi* muscle of Hu sheep was significantly decreased after supplementation with MT. Appiah et al. [43] demonstrated that adding 0.25 mg/mL of MT to sperm diluent significantly increased GSH-Px, CAT, and SOD activities in rooster sperm, while 0.5 mg/mL of MT elevated GSH-Px activity and reduced the MDA content. She et al. [44] observed that MT injections (0.01 g/L) in Chinese mitten crabs significantly lowered MDA levels in muscle and hemolymph 4 h post-injection, with lower-dose groups (0.001 and 0.0001 g/L) showing elevated SOD activity compared to the controls. Supplementation with RPT 5-HTP and MT significantly reduced the MDA content in the *longissimus dorsi* muscle of Hu sheep, and the CAT activity in the *longissimus dorsi* muscle of Hu sheep was significantly increased in the MT group. Sun et al. [30] found no significant differences in plasma T-AOC, MDA, SOD, GSH-Px, or CAT between 5-HTP-supplemented sheep and controls, potentially due to differences in feeding methods/dosages. These results differ from those of the present study, potentially due to variations in feeding protocols and dosage regimens. In our experimental design, RPT 5-HTP was utilized to ensure bypass of the rumen, thereorted that increased dietary tryptophan in quails reduced lipid peroxidation, evidencby facilitating direct delivery to intestinal absorption sites. Sabbaghi et al. [45] reped by decreased MDA in breast meat. Ashok Kumar et al. [46] showed that subcutaneous MT injections (18 mg/kg BW) in water buffalo significantly enhanced serum T-AOC while reducing MDA and nitric oxide levels. Kanyar et al. [47] demonstrated in their study that the MDA content in the *longissimus dorsi* muscle was significantly reduced on day 7 post-slaughter when lambs were implanted with MT (18 mg/head and 36 mg/head). Consistency was observed between the aforementioned studies and the present research findings. In our study, after Hu sheep were supplemented with RPT 5-HTP and MT, a significant reduction in the MDA content in the *longissimus dorsi* muscle was observed in both the 5-HTP and MT groups, while CAT activity in the *longissimus dorsi* muscle was significantly increased in the MT group. These findings indicate that MT and its precursors can enhance the antioxidant capacity of animals.

Meat color is the most intuitive indicator for evaluating meat quality. Among these parameters, a* primarily reflects the myoglobin content in muscle and is positively correlated with meat color redness; L* represents muscle brightness, where lower L* values indicate better meat color; and b* is correlated with meat freshness, showing an inverse relationship with freshness [48]. Muscle pH directly influences tenderness and juiciness, serving as a comprehensive indicator in meat quality assessment. Tenderness, a critical indicator of meat quality and juiciness, is inversely related to shear force, where the lower the shear force, the greater the tenderness. This study showed that supplementation with RPT 5-HTP and MT had no significant effect on the shear force and pH value of the *longissimus dorsi* muscle in Hu sheep, indicating that 5-HTP and MT have no effect on the tenderness of mutton. Chen et al. [49] observed that MT supplementation in broiler diets had no significant effects on carcass traits, pH, drip loss, or cooking loss, but a* and b* values 15 min post-slaughter in the 80 mg/kg MT group were significantly lower than the control. Song et al. [50] found that intramuscular vitamin A injections in newborn lambs improved muscle redness, crude protein, crude fat, and antioxidant capacity, thereby enhancing lamb meat quality. In our study, supplementary feeding of RPT 5-HTP and MT had no effect on the L*, a*, and b* values of the *longissimus dorsi* muscle in Hu sheep. Cooking loss and drip loss are associated with moisture content in muscle tissue and the difficulty of water separation from the muscle matrix, reflecting changes in water-holding capacity [51]. Tian et al. [52] found in their study on grape pomace extract supplementation in finishing pig diets that T-AOC and SOD activity in the longissimus thoracis muscle significantly increased, while drip loss and cooking loss significantly decreased, with pork quality being improved through enhanced antioxidant capacity in finishing pigs. Tian et al.’s research is consistent with the results of this study. In our study, the dietary supplementation of both RPT 5-HTP and MT significantly reduced the drip loss of *longissimus dorsi* muscle in Hu sheep, thereby improving mutton quality.

A higher content of umami amino acids in the same food enhances flavor quality. Six umami amino acids, namely glycine, isoleucine, proline, serine, alanine, and glutamic acid, are essential precursors for meat flavor formation and directly influence meat characteristics, with glutamic acid being the predominant umami compound that contributes to savory taste and mitigates adverse flavors such as saltiness and sourness. This study demonstrated that dietary supplementation of RPT 5-HTP and MT significantly increased the glutamate content in the *longissimus dorsi* muscle of Hu sheep, indicating that MT and 5-HTP have the ability to buffer the salty and sour tastes of mutton, thereby improving mutton taste. Essential amino acids are critical for human growth and metabolism as they cannot be synthesized endogenously and must be obtained through dietary intake [53]. Aspartic acid and glutamic acid are well known for their remarkable mellow and umami characteristics. In our study, dietary supplementation with RPT 5-HTP and MT significantly increased the glutamic acid content in the *longissimus dorsi* muscle of Hu sheep, indicating that both MT and 5-HTP have the ability to enhance the mellow and umami flavors of mutton. Alanine and glycine can stimulate the taste buds to produce a sweet sensation and counteract the bitterness and saltiness of amino acids [54]. Notably, 5-HTP and MT significantly increased the alanine content in the *longissimus dorsi* muscle, suggesting that both MT and 5-HTP can enhance the sweet taste of mutton. In our study, the group supplemented with RPT 5-HTP also showed significant increases in the contents of serine, valine, threonine, isoleucine, phenylalanine, tyrosine, proline, leucine, and methionine in the *longissimus dorsi* muscle of sheep. Supplementation with MT significantly increased the contents of leucine, serine, valine, threonine, isoleucine, phenylalanine, tyrosine, proline, methionine, arginine, and histidine in the *longissimus dorsi* muscle of Hu sheep. The contents of umami amino acids and sweet amino acids in the *longissimus dorsi* muscle of Hu sheep in the experimental groups were significantly increased, indicating that MT and 5-HTP play a role in improving the palatability and flavor of mutton.

The fatty acid composition and content in muscle directly influence lamb meat quality and nutritional value while also serving as critical precursors of meat flavor [55]. Saturated fatty acids are often considered undesirable for our health [56]. Studies have established a strong positive correlation between saturated fatty acid intake and the prevalence of cardiovascular diseases [57]. Therefore, it is particularly important to reduce the content of saturated fatty acids in mutton. Notably, C18:0 levels in saturated fats exhibit a positive correlation with the off-flavor intensity in lamb, where a higher C18:0 content corresponds to stronger undesirable odors [58]. Through supplementation with RPT 5-HTP and MT, we found that the C18:0 content in the *longissimus dorsi* muscle of Hu sheep was significantly reduced, this indicates that MT and 5-HTP have the potential to mitigate mutton off-flavor. Monounsaturated fatty acids, such as oleic acid, and polyunsaturated fatty acids, such as linoleic acid and linolenic acid, are essential fatty acids for human health as they regulate lipid metabolism, lower the levels of cholesterol and triglycerides in the blood, and help prevent cardiovascular diseases [59]. The results of this trial showed that the content of C18:1n9c in the *longissimus dorsi* muscle of Hu sheep in the RTP 5-HTP group was significantly reduced, while there were no significant differences in the contents of C18:2n6c, C18:3n6, and C18:3n3 among groups. Studies have shown that linolenic acid (ALA, n-3) and long-chain polyunsaturated fatty acids n-3, such as eicosapentaenoic acid (C20:5n3) and docosahexaenoic acid (C22:6n3), have potential benefits for heart health [60]. In our study, there was no significant difference in the C20:5n3 content in the *longissimus dorsi* muscle of Hu sheep among groups. In conclusion, 5-HTP and MT can improve the taste of mutton, but they have little benefits for physical health.

Antioxidant indicators and antioxidant enzyme activities in animal products are closely associated with product shelf life and quality, where higher antioxidant enzyme activity is correlated with extended shelf life [3]. MT has natural antioxidant capacity. Under oxidative stress conditions, an antioxidant cascade is formed by MT and its metabolites, resulting in the generation of free radical scavengers that directly counteract highly toxic ROS [61]. Additionally, MT indirectly enhances endogenous antioxidant enzyme activity and improves the efficacy of other antioxidants to protect tissues and cellular structures, thereby inhibiting oxidative damage [17]. TVB-N, a biomarker for protein and amine degradation, arises from the breakdown of proteins, amines (particularly trimethylamine oxide (TMAO), trimethylamine (TMA), dimethylamine (DMA), and formaldehyde), and adenine nucleotide deamination. Its formation is linked to endogenous enzymes and spoilage bacterial activity on methylated amines, making TVB-N a key indicator of meat freshness. During the storage of mutton, its proteins are decomposed by microorganisms and endogenous proteases. The TVB-N content typically increases with the prolongation of meat storage time, and this increase is consistent with other spoilage biomarkers. Therefore, TVB-N values are usually used to determine the freshness and quality of protein-rich foods. The higher the TVB-N value, the higher the degree of spoilage and deterioration of meat products [62]. According to GB 2707-2016 [24], a TVB-N threshold of 15 mg/100 g serves as a shelf-life criterion. Chikwanha et al. [63] demonstrated that grape pomace supplementation in diets enhanced antioxidant capacity in lamb, delaying lipid and protein oxidation and extending shelf life. This study utilized RPT 5-HTP and MT supplementation to prolong lamb shelf life, aligning with Chikwanha’s findings. The results show that the RPT 5-HTP and MT groups significantly reduced the MDA content in the *longissimus dorsi* muscle and extended the shelf life, with the MT group outperforming the RPT 5-HTP group. The RPT 5-HTP group extended the shelf life by 22.08 h, while the MT group extended it by 45.36 h. This suggests that MT has the potential to reduce the MDA content in mutton and extend its shelf life. The shelf-life duration is strongly correlated with the tissue’s antioxidant capacity, which is essentially consistent with the findings reported in the aforementioned literature.

A limitation of the present study was that the mechanisms through which 5-HTP and MT improve mutton quality, extend the shelf life, and enhance the amino acid content were not thoroughly investigated. In terms of molecular mechanisms, there are certain limitations in the exploration, as high-throughput technical methods such as transcriptomics and proteomics were not fully integrated, thus failing to systematically reveal the key gene networks and signaling pathway mechanisms by which dietary supplementation with RPT 5-HTP and MT regulates the quality and shelf life of Hu sheep meat.

## 5. Conclusions

This study shows that dietary supplementation with RPT 5-HTP and MT in the experimental groups significantly reduced the contents of MDA, drip loss, and TVB-N in the *longissimus dorsi* muscle of Hu sheep and significantly increased the contents of most amino acids in the *longissimus dorsi* muscle. Among them, MT significantly increased CAT activity in the *longissimus dorsi* muscle of Hu sheep. This indicates that 5-HTP and MT can enhance the antioxidant capacity of sheep to improve mutton quality and improve the umami characteristic and flavor of mutton by increasing the amino acid content in mutton, and both can prolong the shelf life of mutton.

## Figures and Tables

**Table 1:** Composition and nutrient levels of basal diets (DM basis).

Ingredients	Content (%)	Nutrient Levels	Content (%)
Corn silage	29.31	Neutral detergent fiber	42.38
Corn	26.79	Acid detergent fiber	25.76
Tofu pulp	15.00	Crude protein	12.99
Cotton hull	10.00	Ash	4.35
Cottonseed meal	7.60	Ether extract	3.29
Soybean meal	2.74	Calcium	0.68
Rapeseed meal	2.35	Phosphorus	0.33
Linseed meal	1.72		
Wheat bran	1.72		
Lysine	1.58		
Premix ^1^	0.84		
NaCl	0.35		
Total	100.00		

^1^ Note: Premix for each kilogram of diet: vitamin A, 4200 IU; vitamin B1, 0.4 mg; vitamin B2, 2 mg; vitamin B6, 1.2 mg; vitamin C, 20 mg; vitamin D3, 880 IU; vitamin E, 500 lU; pantothenic acid, 10 mg; copper, 25 mg; iron, 107 mg; manganese, 81 mg; zinc, 74 mg; iodine, 6 mg; selenium, 14 mg; cobalt, 3 mg; nicotinamide, 100 mg; choline chloride, 120 mg.

**Table 2:** Effects of RPT 5-HTP and MT supplementation on growth performance of Hu sheep.

Items	Control Group	RPT 5-HTP Group	MT Group	SEM	*p*-Value
Dry matter intake (kg/d)	1.15	1.16	1.16	0.023	0.982
Initial body weight (kg)	30.91	30.83	30.79	0.212	0.974
Final body weight (kg)	35.85	35.93	36.01	0.306	0.978
Average daily gain (g/d)	164.50	170.00	174.00	5.128	0.770
Ratio of feed intake to gain	7.10	6.88	6.72	0.102	0.329

**Table 3:** Effects of 5-HTP and MT supplementation on slaughter performance of Hu sheep.

Items	Control Group	RPT 5-HTP Group	MT Group	SEM	*p*-Value
Greyhound rib (mm)	12.15	12.42	12.80	0.248	0.589
Eye muscle area (cm^2^)	18.14	18.14	19.76	0.345	0.079
Live weight (kg)	36.12	36.18	36.12	0.404	0.997
Carcass weight (kg)	18.45	18.67	18.63	0.146	0.913
Slaughter rate (%)	51.12	51.61	51.57	0.146	0.277

**Table 4:** Effects of RPT 5-HTP and MT supplementation on antioxidation of *longissimus dorsi* muscle in Hu sheep.

Items	Control Group	RPT 5-HTP Group	MT Group	SEM	*p*-Value
Superoxide dismutase U/mg	224.9	221.78	225.06	4.146	0.952
Malondialdehyde nmol/mg	1.44 ^a^	1.12 ^b^	1.12 ^b^	0.058	0.020
Glutathione peroxidase U/mg	130.14	123.72	129.60	3.712	0.805
Catalase U/mg	0.39 ^b^	0.45 ^ab^	0.50 ^a^	0.012	0.009
Total antioxidant capacity mmol/g	0.08	0.08	0.07	0.002	0.404

In the same row, values with no letter or the same letter superscripts mean no significant difference (*p* > 0.05), while with different letter superscripts mean significant difference (*p* < 0.05).

**Table 5:** Effects of RPT 5-HTP and MT supplementation on meat quality of *longissimus dorsi* muscle in Hu sheep.

Items	Control Group	RPT 5-HTP Group	MT Group	SEM	*p*-Value
pH_45min_	6.31	6.19	6.22	0.059	0.700
Brightness L*	34.16	34.22	33.44	0.411	0.717
Infrared a*	14.99	15.27	13.93	0.354	0.278
Yellowness b*	5.97	5.95	5.73	0.110	0.642
pH_24h_	5.84	5.81	5.71	0.030	0.177
Shear force (N)	45.85	45.76	45.98	0.266	0.950
Drip loss (%)	11.84 ^a^	9.64 ^b^	7.83 ^c^	0.441	<0.01
Cooking loss (%)	39.38	37.25	38.37	0.522	0.264

In the same row, values with no letter or the same letter superscripts mean no significant difference (*p* > 0.05), while with different letter superscripts mean significant difference (*p* < 0.05).

**Table 6:** Effects of RPT 5-HTP and MT supplementation on amino acid content in *longissimus dorsi* muscle of Hu sheep (ug/mg).

Items	Amino Acid	Control Group	RPT 5-HTP Group	MT Group	SEM	*p*-Value
Essential amino acids	Trp	0.11	0.18	0.19	0.020	0.278
	Lysine	13.88	13.15	14.32	0.344	0.399
	Leucine	18.76 ^c^	22.13 ^b^	24.43 ^a^	0.690	<0.01
	Histidine	3.58 ^b^	3.71 ^ab^	4.22 ^a^	0.116	0.046
	Methionine	5.47 ^c^	6.72 ^b^	7.62 ^a^	0.268	<0.01
	Valine	12.50 ^c^	14.75 ^b^	16.25 ^a^	0.442	<0.01
	Threonine	7.05 ^c^	9.21 ^b^	11.22 ^a^	0.453	<0.01
	Isoleucine	11.46 ^c^	13.73 ^b^	15.10 ^a^	0.429	<0.01
	Phenylalanine	8.75 ^b^	10.38 ^a^	11.27 ^a^	0.312	<0.01
Non-essential amino acids	Glycine	3.03	3.02	2.53	0.126	0.192
	Alanine	14.18 ^c^	18.27 ^b^	21.07 ^a^	0.782	<0.01
	Tyrosine	6.74 ^b^	8.24 ^a^	8.96 ^a^	0.291	<0.01
	Proline	11.15 ^c^	13.63 ^b^	15.10 ^a^	0.463	<0.01
	Aspartic acid	6.4	5.08	5.80	0.279	0.142
	Glutamic acid	22.22 ^c^	27.82 ^b^	33.68 ^a^	1.242	<0.01
	Serine	3.16 ^c^	6.91 ^b^	9.30 ^a^	0.739	<0.01
	Arginine	8.55 ^b^	9.73 ^b^	11.75 ^a^	0.393	<0.01
Essential amino acids		81.55 ^c^	93.96 ^b^	104.62 ^a^	2.722	<0.01
Non-essential amino acids		75.46 ^c^	92.70 ^b^	108.20 ^a^	3.579	<0.01
Umami amino acids		65.20 ^c^	83.38 ^b^	96.79 ^a^	3.507	<0.01
Sweet amino acids		61.35 ^c^	72.81 ^b^	83.32 ^a^	2.403	<0.01

The sweet amino acids in the table are serine, threonine, glycine, alanine, and proline; umami amino acids include glycine, isoleucine, proline, serine, alanine, and glutamic acid. In the same row, values with no letter or the same letter superscripts mean no significant difference (*p* > 0.05), while with different letter superscripts mean significant difference (*p* < 0.05).

**Table 7:** Effects of 5-HTP and MT supplementation on fatty acid content in *longissimus dorsi* muscle of Hu sheep (ug/100 mg).

Items	Control Group	RPT 5-HTP Group	MT Group	SEM	*p*-Value
C4:0	0.037	0.042	0.045	0.004	0.816
C6:0	0.026	0.021	0.021	0.003	0.697
C8:0	0.180	0.126	0.135	0.021	0.569
C10:0	1.567	1.152	1.040	0.244	0.667
C11:0	0.055	0.036	0.051	0.005	0.217
C12:0	0.848	0.498	0.465	0.130	0.436
C13:0	0.071	0.037	0.045	0.009	0.326
C14:0	24.371	15.189	15.280	3.778	0.551
C15:0	2.628	1.412	1.844	0.336	0.346
C16:0	329.631	220.571	229.061	42.503	0.536
C17:0	12.026	6.755	7.645	1.544	0.349
C18:0	204.886 ^a^	97.632 ^b^	110.154 ^ab^	18.350	0.022
C20:0	0.602	0.393	0.285	0.103	0.469
C21:0	0.100	0.105	0.119	0.004	0.129
Saturated Fatty Acids	577.03	343.97	366.19	63.886	0.273
C14:1	0.765	0.536	0.649	0.095	0.647
C16:1	17.706	12.052	14.900	2.435	0.665
C18:1n9c	439.291 ^a^	265.987 ^ab^	228.701 ^b^	34.518	0.018
C20:1	0.886	0.689	0.647	0.083	0.485
Monounsaturated Fatty Acids	458.65 ^a^	279.26 ^b^	244.90 ^b^	36.328	0.024
C18:2n6c	89.019	66.251	59.555	7.121	0.217
C18:3n6	0.529	0.408	0.269	0.066	0.294
C18:3n3	2.736	1.884	1.521	0.299	0.247
C20:2	2.114 ^Aa^	1.840 ^Aab^	1.326 ^Ab^	0.076	0.076
C20:3n6	1.391	1.277	1.153	0.068	0.377
C20:4n6	16.685	17.688	14.728	0.847	0.371
C20:5n3	0.482	0.535	0.429	0.023	0.161
C22:6	0.489	0.555	0.497	0.018	0.268
Polyunsaturated Fatty Acids	204.89	200.36	102.55	35.324	0.216

In the same row, values with no letter or the same letter superscripts mean no significant difference (*p* > 0.05), while with different letter superscripts mean significant difference (*p* < 0.05).

## Data Availability

The data that support the findings of this study are available from the corresponding author upon reasonable request.
